# Evaluation of the 10 Warning Signs in Primary and Secondary Immunodeficient Patients

**DOI:** 10.3389/fimmu.2022.900055

**Published:** 2022-05-13

**Authors:** Fadime Ceyda Eldeniz, Yahya Gul, Alaaddin Yorulmaz, Sukru Nail Guner, Sevgi Keles, Ismail Reisli

**Affiliations:** ^1^Department of Pediatrics, Necmettin Erbakan University, Konya, Turkey; ^2^Department of Pediatric Allergy and Immunology, Necmettin Erbakan University, Konya, Turkey; ^3^Department of Pediatrics, Selcuk University, Konya, Turkey

**Keywords:** primary immunodeficiency, secondary immunodeficiency, combined immunodeficiency, 10 warning signs, childhood

## Abstract

**Objectives:**

Ten warning signs of primary immunodeficiency (PID) were suggested by the Jeffrey Modell Foundation (JMF), to increase physician awareness of PID. These warning signs have not yet been evaluated for patients with secondary immunodeficiency (SID). This study investigated whether the 10 warning signs used for the diagnosis of PID were also sufficient for the diagnosis of SID, and explored the possibility of additional signs.

**Methods:**

This prospective study was conducted between June and December 2020. The mothers of 162 patients with PID and SID, and mothers of 200 healthy children, were asked to complete a questionnaire about family and personal history in addition to the warning signs of PID developed by the JMF. A JMF score was created by giving one point for each “Yes” answer for the 10 warning signs of PID. Medical records of the patients were evaluated for possible additional warning signs for PID and SID.

**Results:**

The JMF scores of the PID (3.36 ± 1.65) and SID (3.72 ± 1.12) groups were significantly higher than the scores of the control group (0.34 ± 0.61) (p < 0.05). A sign for immunological evaluation in two patients without warning signs in the PID group was found to be chronic diarrhea. In addition to the 10 JMF warning signs, we found that consanguinity and a family history of tuberculosis were statistically significant in our PID group, compared with the SID and control groups.

**Conclusions:**

The JMF warning signs are important for early diagnosis of PID. Our study showed that these signs may also be used for the early diagnosis of SID in patients and, according to our results, in addition to the 10 JMF signs for PID, parental consanguinity, chronic diarrhea, and a family history of tuberculosis may also be considered warning signs for the early diagnosis of PID.

## Introduction

Primary immunodeficiencies (PIDs) are a group of diseases comprised of more than 450 innate errors of immunity (IEI), and they are becoming more prevalent (1). Although PIDs are rare diseases, they are more common than previously thought, following the use of modern diagnostic methods (2). A recent evaluation indicated that at least 1–2% of the world’s population are affected by PIDs (3). Studies from our country also reveal a high incidence of PIDs in children. In this regard, training family physicians, pediatricians and, in particular, infectious disease specialists about PIDs will allow early diagnosis of these patients; early and effective treatment may allow them to reach adulthood (4, 5).

Several warning signs have been developed to increase physician awareness about the early diagnosis of PIDs. Warning signs of PID were developed by an institution called the Jeffrey Modell Foundation (JMF), which tries to increase awareness of this issue (http://www.info4pi.org/library/educational-materials/10-warning-signs). A detailed history should be taken for children admitted with a history of frequent infections, and the 10 warning signs of PID described by the JMF should be evaluated in addition to a full physical examination. This approach will allow early diagnosis of PID patients and, hence, the possibility of early and effective treatment before the development of organ damage (6, 7).These warning signs have not yet been evaluated for patients with secondary immunodeficiency (SID).

The objective of this study was to investigate whether the 10 warning signs used for the diagnosis of PID are sufficient for the diagnosis of SID, and to explore the possibility of additional signs.

## Materials and Methods

This prospective study was conducted between June and December 2020. This research was conducted using data obtained for clinical purposes. The study was approved by Necmettin Erbakan University Meram Medical School Ethics Committee (Date: 06.26.2020/No: 2020/2599).

The mothers of 162 patients diagnosed with PID and SID were asked to complete a questionnaire about family and personal history, in addition to the 10 warning signs of PID developed by the JMF. The same survey was completed by the mothers of 200 children without any defined primary/secondary immunodeficiency (i.e., healthy), and they formed the control group.

The study group was divided into two groups based upon clinical and laboratory findings to form the PID and SID groups. In addition to the questionnaire, the medical records of patients in the study groups were evaluated; age at diagnosis, treatment of the immunodeficiency, duration of diagnostic delay, and patient characteristics during the follow-up period were recorded. The SID was defined as using ESID criteria and the patients with chromosomal anomaly (50%), anti-epileptic drug use (30%),malnutrition 10%, uremia (5%), metabolic disease (5%) were classified as SID group. The questionnaire was provided to mothers of the study and control groups and the responses were recorded by the same research scientist. A JMF score was created by giving one point for each “Yes” answer for the 10 warning signs of PID.

### Statistical Analyses

Analysis of the study data was performed by using the SPSS 25 program (IBM Corp. Released 2017. IBM SPSS Statistics for Windows, Version 25.0. Armonk, NY, USA). Frequency, ratio, mean, and standard deviation (SD) of different variables of the individuals were analyzed by descriptive statistics. The Independent Samples T-test was used for dual comparisons, whereas triple comparisons were performed using one-way analysis of variance. Mean ± SD values of the groups were reported for evaluation of distribution rates of different variables by the groups using chi-square analysis. Crosstabs were made, and numbers and ratios were reported in the crosstabs. For variables found to be significant as a result of chi-square analyses performed for triple groups, further dual comparisons were performed to find out the reason for this difference. The level of significance was determined to be p < 0.05.

## Results

Of the patients included in the study, there were 98 (27.1%) in the PID group, and 64 (17.7%) in the SID group; the control group consisted of 200 (55.2%) healthy children. Overall, there were 200 (55.2%) males and 162 (44.8%) females. In the PID group, 48 (49%) were female and 50 (51%) were male, whereas in the SID group, 16 (25%) were female and 48 (75%) were male. In the control group, 98 (49%) were female and 102 (51%) were male. When sex distribution ratios were analyzed by group, the ratio of males in the SID group was significantly higher, compared with the PID and control groups (p < 0.001).

Ages of the participants varied between 1–216 months (mean ± SD: 74.93 ± 62.59). The mean age was 98.87 ± 66.37 months in the PID group, 67.89 ± 40.19 months in the SID group, and 65.46 ± 63.71 months in the control group. The demographic data of the patients are shown in **Table 1**. The mean age of the PID group was significantly higher compared with that of the control and SID groups (p < 0.001). There was no statistical difference between the control and SID groups.

**Table 1:** The demographic characteristics of the patients with PID and SID, and the control group.

	Control		PID		SID		p
	Mean±SD	Median (min-max)	Mean±SD	Median (min-max)	Mean±SD	Median (min-max)	
**Age (Month)**	65.46±63.71	42 (1-204)	98.87±66.37	84 (7-216)	67.89±40.19	57 (3-204)	0.001
**Age at first hospitalization (Month)**	19.30±23.29	12.0 (1-120)	14.62±26.66	3.0 (1-162)	7.87 ±13.80	2.0 (1-60)	0.001
**Age at diagnosis of immunodeficiency (Month)**			48.58±53.74	21.00 (1-192)	36.64±38.87	24.00 (2-174)	0.127
**Duration of diagnostic delay (Month)**			21.04±32.71	10.50 (1-186)	21.67±25.79	12.0 (1-108)	0.897
**Age at initiation of treatment (Month)**			55.06±58.36	27.00 (1-210)	37.73±38.92	24.00 (2-174)	0.025
**Duration of treatment (Month)**			39.22±32.42	30.00 (1-162)	28.89±21.03	24.00 (1-120)	0.015
**Age at discontinuation of treatment (Month)**			97.50±60.57	84.00 (36-204)	39.00±4.24	39.00 (36-42)	0.228
**Duration of follow-up (Month)**			49.57±41.76	36.00 (5-192)	34.00±22.95	30.00 (1-120)	0.007

PID, primary immunodeficieny; SID, secondary immunodeficiency.

When the PID and SID groups were compared, age at initiation of therapy in the PID group was older (p < 0.05), and the duration of therapy and follow-up period were longer (p < 0.05) in the PID group compared with those of the SID group, with statistically significant differences.

JMF scores of the PID (3.36 ± 1.65) and SID (3.72 ± 1.12) groups were significantly higher than those of the control group (0.34 ± 0.61) (p < 0.001). The distribution of JMF scores of the PID, SID, and control groups is shown in **Table 1E**. The JMF score was zero in two patients in the PID group; these two patients had been screened for PID due to chronic diarrhea. The IUIS classification (2020) (8) of patients with PID, and the distribution of JMF scores of the PID subgroups are shown in **Table 2E**.

**Table 1E:** Distribution of JMF scores of the PID, SID and Control Groups.

JMF Score	PID Group (n/%)		SID Group (n/%)			Control Group (n/%)			Total (n/%)	
**0 point**	2 (2.0%)		0			146 (73.0%)			148 (40.9%)	
**1 point**	10 (10.2%)		1 (1.6%)			41 (20.5%)			52 (14.4%)	
**2 points**	23 (23.5%)		8 (12.5%)			12 (6.0%)			43 (11.9%)	
**3 points**	17 (17.3%)		18 (28.1%)			1 (0.5%)			36 (9.9%)	
**4 points**	22 (22.4%)		21 (32.8%)			0			43 (11.9%)	
**5 points**	14 (14.3%)		13 (20.3%)			0			27 (7.5%)	
**6 points**	6 (6.1%)		3 (4.7%)			0			9 (2.5%)	
**7 points**	4 (4.1%)		0			0			4 (1.1%)	
**8 points**	0		0			0			0	
**9 points**	0		0			0			0	
**10 points**	0		0			0			0	

JMF, Jeffrey Modell Foundation.

After evaluation of overall JMF scores, answers given to the questions were also compared separately. Regarding having ≥ 4 episodes of otitis in one year, ≥ 2 episodes of sinusitis in one year, and a family history of PID, the rates in the PID group were significantly higher than the control and SID groups (p < 0.001). No difference was found between the control and SID groups. Regarding oral antibiotic use for ≥ 2 months with little effect, recurrent deep tissue infections or organ abscesses, persistent thrush or cutaneous fungal infections, and ≥ 2 deep tissue infections including septicemia, the rates in the PID and SID groups were significantly higher compared with the control group (p < 0.001). No difference was found between the PID and SID groups. Regarding failure to thrive, the need for IV antibiotics to clear infections, and having ≥ 2 lower respiratory tract infections in one year, the rates in the SID group were higher than the PID group. The rate in the PID group was significantly higher than the control group (p < 0.001). Distribution of the 10 warning signs by groups is shown in **Table 2**.

**Table 2:** Distribution of the warning signs of the groups according to Jeffrey Modell Foundation.

Criteria			PID		SID		Control Group		*P*
			N	%	n	%	n	%	
Oral antibiotic	Two or more months on antibiotics with little effect.	No	53	54.1	33	51.6	192	96	<0.001
		Yes	45	45.9	31	48.4	8	4	
Abscess	Recurrent, deep skin or organ abscesses	No	89	90.8	60	93.8	199	99.5	<0.001
		Yes	9	9.2	4	6.3	1	0.5	
Growing	Failure of an infant to gain weight or grow normally	No	61	62.2	29	45.3	197	98.5	<0.001
		Yes	37	37.8	35	54.7	3	1.5	
Thrush	Persistent thrush in mouth or fungal infection on skin	No	55	56.1	34	53.1	188	94	<0.001
		Yes	43	43.9	30	46.9	12	6	
IV antibiotic	Need for intravenous antibiotics to clear infections	No	17	17.3	0	0	170	85	<0.001
		Yes	81	82.7	64	100	30	15	
Septicemia	Two or more deep-seated infections including septicemia	No	88	89.8	55	85.9	200	100	<0.001
		Yes	10	10.2	9	14.1	0	0	
PI in family	A family history of PI	No	73	74.5	60	93.8	196	98	<0.001
		yes	25	25.5	4	6.3	4	2	
Ear infection	Four or more new ear infections within 1 year	No	90	91.8	63	98.4	199	99.5	<0.001
		Yes	8	8.2	1	1.6	1	0.5	
Sinus infection	Two or more serious sinus infections within 1 year	No	89	90.8	63	98.4	198	99	<0.001
		Yes	9	9.2	1	1.6	2	1	
Pneumonia	Two or more pneumonias within 1 year	No	35	35.7	5	7.8	193	96.5	<0.001
		Yes	63	64.3	59	92.2	7	3.5	

Answers of the PID group to questions regarding the 10 warning signs of PID were estimated as observed and expected values. The criteria that exhibited a significant difference included the rates of recurrent deep cutaneous or organ abscesses, failure to thrive, need for IV antibiotics, ≥ 2 deep tissue infections including septicemia, a family history of PID, ≥ 4 episodes of otitis in one year, ≥ 2 episodes of sinusitis in one year, and ≥ 2 lower respiratory tract infections in one year. The criteria that exhibited the least differences between expected and observed values included the rates of being on oral antibiotics for ≥ 2 months with little effect, and a history of persistent thrush or cutaneous fungal infections. Among the warning signs of PID developed by the JMF, the criteria with the most statistically significant differences included the rates of ≥ 4 episodes of otitis in one year, recurrent deep cutaneous or organ abscesses, ≥ 2 episodes of sinusitis in one year, and ≥ 2 deep tissue infections including septicemia. These four warning signs seem to be less indicative of PID according to the statistics.

Odds ratios (ORs) for the 10 warning signs were estimated for both PID and SID groups versus the control group. Being on oral antibiotics was increased by 20.37-fold in the PID group compared with the control group, with a sensitivity of 45.91% and specificity of 96.00%. In the SID group, being on oral antibiotics was increased by 22.54-fold, with a sensitivity of 48.43% and specificity of 96.00%. OR, sensitivity, and specificity of the 10 warning signs are shown in **Table 3**.

**Figure 1:**
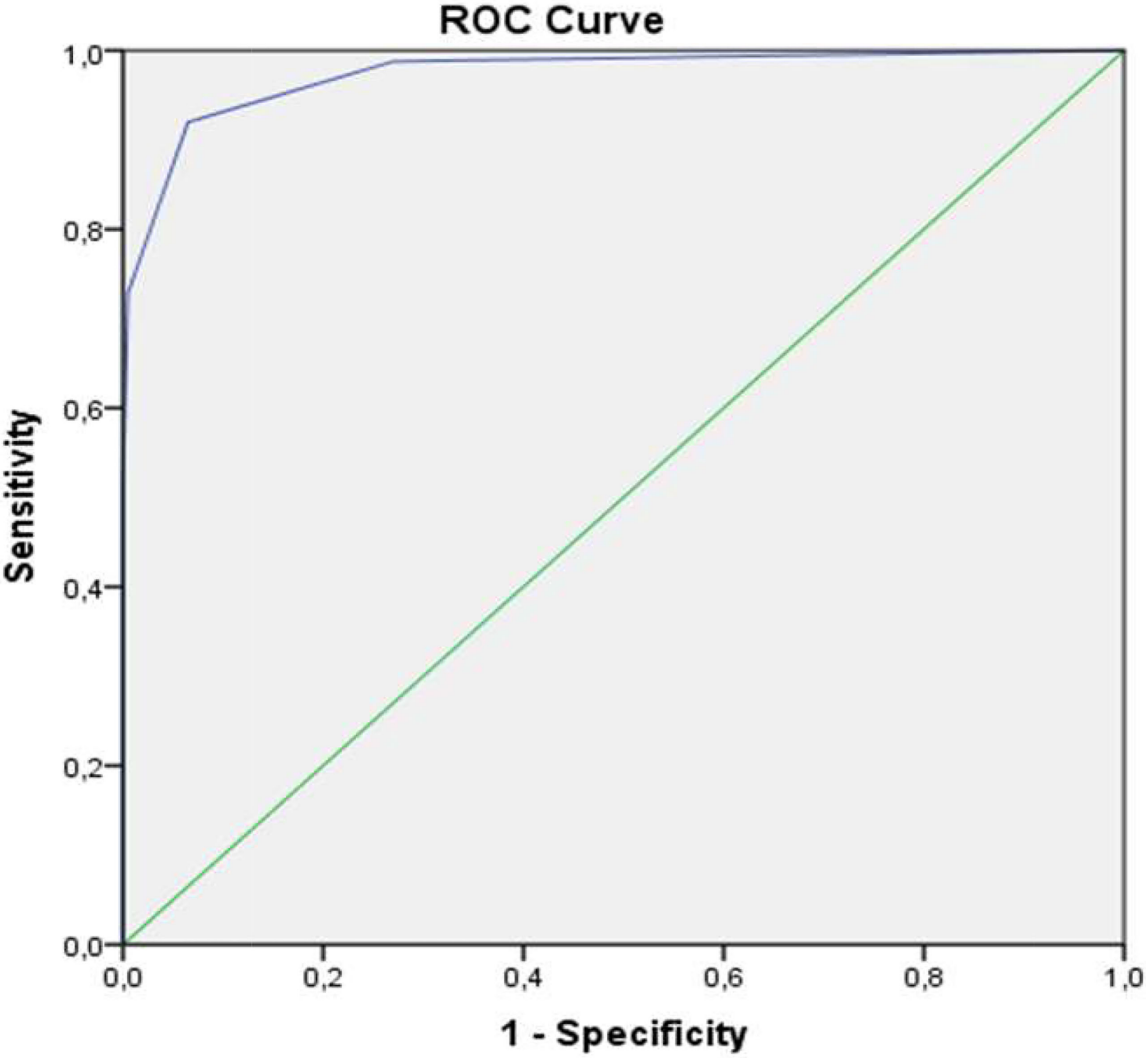
The ROC curve for JMF score in the PID and SID groups.

According to the receiver operating characteristic (ROC) results, the predictive area under the ROC curve (AUC) value for the JMF score in the PID and SID groups was determined to be 0.974 (95% confidence interval (CI), 0.959–0.989), which was statistically significant (p < 0.001). The ROC curve for the JMF score in the PID and SID groups is shown in **Figure 1**. The cut-off for the JMF score in the PID and SID groups was determined to be 1.5. In accordance with the determined cut-off, sensitivity was found to be 0.920% and specificity was 0.935%.

When parental consanguinity was examined between the groups, it was revealed that 41 patients (41.8%) in the PID group, 16 patients (25%) in the SID group, and 18 patients (9%) in the control group had consanguineous parents. The distribution of family history features of the patients who participated in the study by groups is shown in **Table 3E**. A statistically significant difference was determined between the groups (p < 0.001). The rate of parental consanguinity of the PID group was higher than both SID and control groups. The rate of parental consanguinity in the PID group was 7.27-fold higher than that of the control group (OR: 7.27; 95% CI: 3.87–13.64). This rate was 3.37-fold higher in the SID group (95% CI: 1.60–7.09).

In both the PID and SID groups, the rates of a family history of early death, rheumatic diseases, and malignancies were higher, compared with the control group (p < 0.05). The rate of a family history of tuberculosis was significantly higher in the PID group, compared with the SID and control groups, whereas the rate of a family history of allergic diseases was significantly higher in the control group compared with the PID and SID groups.

**Figure 2:**
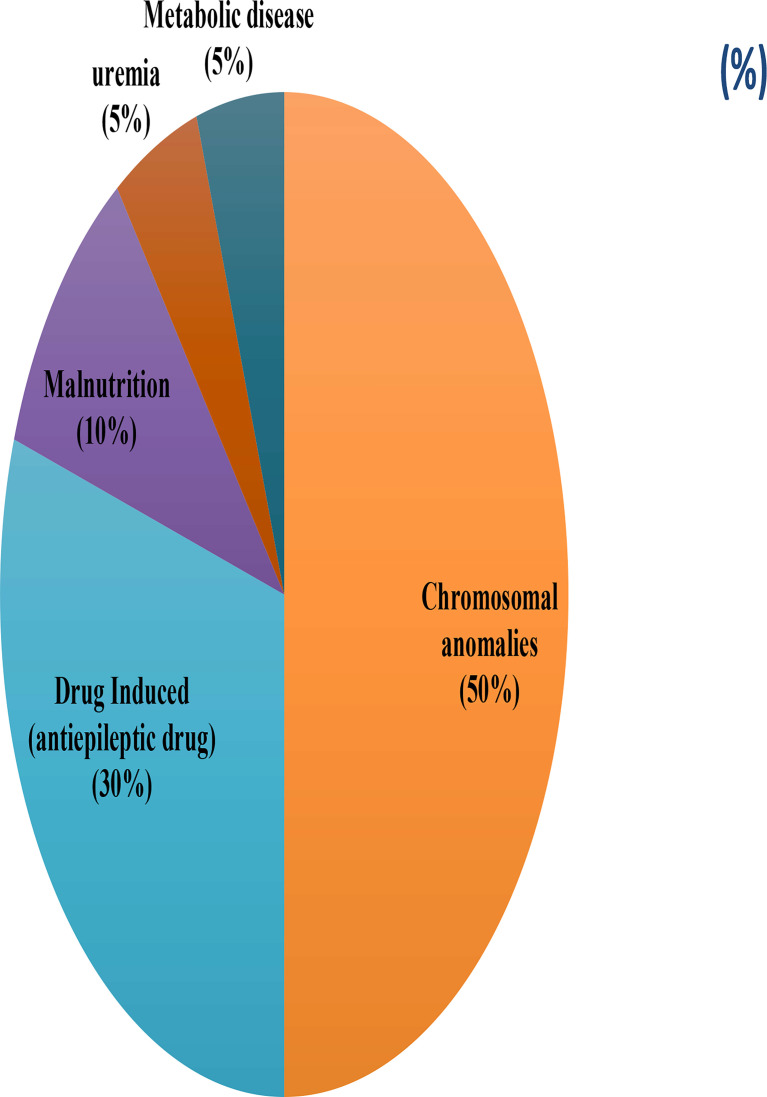
The diagnosis of the patients with SID.

The most common group of diseases in our PID patients was that of immunodeficiencies due to antibody deficiency, as shown in **Table 2E**. Clinical characteristics of the patients with SID are shown in **Figure 2** and **Table 4**.

**Table 2E:** IUIS classification of the patients with PID (2020) and JMF scores of the PID subgroups.

Diagnoses	n	%	JMF scoreMean±SD	JMF scoreMedian (Min-Max)
Immunodeficiencies affecting cellular and humoral immunity	18	18.4	3.28±1.60	3.00 (1-7)
Combined immunodeficiencies with associated syndromic features	21	21.4	3.90±1.44	4.00 (1-6)
Immunodeficiencies due to Antibody Deficiencies	39	39.8	2.95±1.58	2.00 (0-6)
Diseases of immune dysregulation	7	7.1	2.14±1.57	2.00 (0-5)
Congenital defects of phagocyte number or function or both	8	8.2	4.50±1.69	4.00 (2-7)
Defects in Intrinsic and Innate Immunity	1	1	7.00±0.00	7.00 (7-7)
Auto-inflammatory disorders	3	3.1	4.00±0.00	4.00 (4-4)
Complement deficiencies	1	1	3.00±0.00	3.00 (3-3)
Bone marrow failure	0	0.0	.	.
Phenocopies of PID	0	0.0	.	.
**Total**	98	100.0		

IUIS, International Association of Immunology Societies.

**Table 3:** OR, sensitivity and specificity of the 10 warning signs developed by JMF.

		OR (95% CI)	Sensitivity	Specificity	PPV	NPV	Accuracy
Oral antibiotic	**PID**	20.37 (9.05-45.86)	45.91	96.00	84.90	78.36	79.53
	**SID**	22.54 (9.53-53.30)	48.43	96.00	79.48	85.33	84.47
Abscess	**PID**	20.12 (2.51-61.24)	4.32	98.88	90.00	30.90	32.88
	**SID**	13.26 (1.45-120.96)	6.25	99.50	80.00	76.83	76.89
Growing	**PID**	39.83 (11.86-133.71)	37.75	98.50	92.50	76.35	78.52
	**SID**	79.25 (22.89-274.37)	54.68	98.50	92.10	87.16	87.87
Thrush	**PID**	12.24 (6.04-24.83)	43.87	94.00	78.18	77.36	77.51
	**SID**	13.82 (6.44-29.63)	46.87	94.00	71.42	84.68	82.57
IV antibiotic	**PID**	27.00 (14.07-51.78)	82.65	85.00	72.97	90.90	84.22
	**SID**	31.13 (2.33-42.21)	100.00	85.00	68.08	100.00	88.63
Septicemia	**PID**	47.57 (2.75-820.92)	10.10	100.00	100.00	69.20	70.23
	**SID**	68.63 (3.93-197.78)	14.06	100.00	100.00	78.43	79.16
PI in family	**PID**	16.78 (5.64-49.86)	25.51	98.00	86.20	72.86	74.16
	**SID**	3.26 (0.79-13.45)	6.25	98.00	50.00	76.56	75.75
Ear infection	**PID**	17.68 (2.18-143.54)	8.16	99.50	88.88	68.85	69.46
	**SID**	3.15 (0.19-51.23)	1.56	99.50	50.00	75.95	75.75
Sinus infection	**PID**	10.01 (2.12-47.28)	9.18	99.00	81.81	68.99	69.46
	**SID**	1.57 (0.14-17.62)	1.56	99.00	33.33	75.86	75.37
Pneumonia	**PID**	49.62 (21.00-117.26)	64.28	75.39	50.00	84.64	72.31
	**SID**	325.34 (99.56-1063.13)	92.18	96.50	89.39	97.47	95.45

OR, Odds ratio; PPV, Positive predictive value; NPV, Negative predictive value; IV, Intravenous.

**Table 3E:** Distribution of family history features of the patients and the controls by the groups.

	Control Group		PID		SID		Total		P
	N	%	n	%	n	%	n	%	
**Consanguinity**	18	24	41	54.6	16	21.4	75	37.5	<0.001
**First Degree**	14	77.8	33	80.5	10	62.5	57	76	
**Second Degree**	1	5.6	3	7.3	1	6.3	5	6.7	
**Third Degree**	1	5.6	3	7.3	4	25	8	10.7	
**Fourth Degree**	2	11.1	2	4.9	1	6.3	5	6.7	
**Family history of premature death (Yes/No)**	11/189	5.5/94.5	66/32	67.3/32.7	38/26	59.4/40.6	115		<0.001
**Family history of tuberculosis (Yes/No)**	5/195	2.5/97.5	16/82	4.7/95.3	33/61	4.7/95.3	54		<0.001
**Family history of rheumatic diseases (Yes/No)**	23/177	11.5/88.5	28/70	28.6/71.4	22/42	34.4/65.6	73		<0.001
**Family history of allergic diseases (Yes/No)**	98/102	49/51	31/67	31.6/68.4	19/45	29.7/70.3	148		<0.001
**Family history of Malignancy (Yes/No)**	24/176	12/88	28/70	28.6/71.4	19/45	29.7/70.3	71		<0.001

**Table 4:** The diagnosis of the patients with SID.

	N	(%)
**Chromosomal Anomalies**	32	50
** Trisomy 21**	17	
** Unspecified syndromes**	5	
** 46 XX ınv9. XP11 duplıcatıon**	1	
** Brugada syndrome**	1	
** 47 XYY**	1	
** Wıdemann steıner syndrome**	1	
** Klınefelter syndrome**	1	
** Noonan syndrome type 5**	1	
** West syndrome**	1	
** Swyer james syndrome**	1	
** Dravet syndrome**	1	
** Joubert syndrome**	1	
**Drug Induced**	19	30
** Anti-epileptic drug**	19	
**Malnutrition**	7	10
**Metabolic disease**	3	5
** Krabbe disease**	1	
** Nieman pick c disease**	1	
** Metachromatic leukodystrophy**	1	
**Uremia**	3	5

## Discussion

Currently, the awareness level of physicians and healthcare professionals about PID, and their experience with a clinical approach to a patient with PID are still insufficient. For this reason, the history, features, and physical examination findings from patients, as well as expert opinions were combined, and the “10 Warning Signs of Primary Immunodeficiency Diseases”, which has significantly contributed to the diagnosis of PID, was defined by the JMF (9). In our study, the JMF scores of PID and SID patients calculated *via* these warning signs were statistically significantly higher compared with the control group. In addition, we found that parental consanguinity and a family history of tuberculosis, chronic diarrhea may also be warning signs of PID.

Every patient with suspected PID should be asked in detail for information related to the “10 warning signs” checklist during history taking. It has been proposed that PID should be investigated when ≥ 2 warning signs are present (10). In our study, the JMF scores of both the PID and SID groups were significantly higher than the control group. The cut-off for JMF score in terms of PID and SID was determined to be 1.5, with a sensitivity of 92% and specificity of 93.5%. According to our study results, we hypothesize that the JMF criteria are a guiding tool not only for PID patients but also for SID patients. In addition, in a study by Reda et al., at least one of the 10 warning signs was observed in all PID patients, whereas 28% of patients without PID had no warning sign (11). In our study, all patients in the SID group had warning signs of immunodeficiency, whereas two of our patients in the PID group had no warning sign. The reason for these two patients with a JMF score of zero being investigated was a history of chronic diarrhea. Considering this, we propose that a history of chronic diarrhea should be included in the warning signs of PID. We attribute the presence of JMF warning signs in all patients in the SID group to the fact that that they all had a more severe course that required IV immunoglobulin therapy.

Training programs aimed at increasing awareness of PID should target physicians who may discover a family history of PID, parental consanguinity, and a family history of early sibling deaths in societies where consanguineous marriages are common (11). In a study from Egypt by Reda et al., 60% of PID patients had consanguineous parents (11). In our country, the rate of parental consanguinity in PID patients has been found to be 14.3–37.5% (4). In our study, the rate of parental consanguinity in PID cases was 41.8%. This rate was significantly higher compared with the control and SID groups, and we propose that parental consanguinity may be a warning sign of autosomal recessive-inherited PID for our region. According to a study conducted by Subbarayan et al. (6), one of the strongest identifiers of PID was a family history of immunodeficiency. In general, such a family history is 18 times more common in children with PID, compared to those without any identifiable PID. In our study, we determined a family history was 16.78 times more common in the PID patients compared with the control group. We propose that screening for PID would be important in the presence of a family history of PID, even when it exists alone.

In the Subbarayan et al. study, the most common of the 10 warning signs was the need for IV antibiotics. The second most common warning sign was a family history of PID (34%), followed by failure to thrive (31%) (6). Similarly, in the study by Reda et al., the most common warning sign was the need for IV antibiotics (92%). In our study, as in these two studies, the most common warning sign was the need for IV antibiotics (82.9%). The second most common warning sign was having ≥ 2 lower respiratory tract infections in one year (64.3%), and the third was being on oral antibiotics for longer than two months with little effect (45.9%). Our findings confirm that the 10 warning signs may be used for the diagnosis of PID, although in a different order of frequency, and that different frequencies may be reported in different studies.

Frequent infections, a more severe course than expected, long-lasting infections, the occurrence of unexpected or severe complications due to infections, incomplete recovery with antibiotic treatment, the need for prolonged use of antibiotics, chronic courses of infectious diseases, and the occurrence of infections with unusual agents may also be associated with PID diseases (12). Infections usually recover rapidly and without complications in children with a healthy immune system and no other risk factors (13). In our study, among the JMF warning signs, frequent recurrent upper respiratory tract infections were significant in the PID group, and frequent recurrent lower respiratory tract infections and failure to thrive were significant in the SID group. This may be due to the high rate of antibody deficiency, and the presence of accompanying conditions (tracheostomy, epilepsy) in our SID patients.

Comprehensive evaluation of family history and clinical features may be helpful for the early diagnosis of PID disease (14, 15). However, absence of a family history of immunodeficiency does not exclude the presence of PID. Since the majority of PID diseases are inherited, the presence of a similar disease, as well as the age and sex of affected individuals are important. In the study by Yorulmaz et al., 3.8% of PID patients were found to have a family member with PID (4). This was higher in the PID and SID groups in our study, with rates of 25.5% and 6.3%, respectively. These higher rates in our study may be due to asking not only about siblings and parents but also about the siblings of the parents and their children. In our study, the rate in the PID group was significantly higher than both the control and SID groups. A patient with a history of frequent infections and a family history of PID should be evaluated for PID.

In a study conducted by Yorulmaz et al. in Konya, the rate of sibling death among patients with combined immunodeficiency (CID) was 7.5% (4). However, in the Reda study from Egypt, 21.7% of the patients had sibling deaths. This 3-fold higher rate in Egypt may be related to a higher rate of consanguineous marriages and the level of community and economic development. The highest rate of sibling death was determined to be 50% in those with CID (11). Rates of early death in the family history were also evaluated in our study. According to the results, 66 (67.3%) of PID cases, 38 (59.4%) of SID cases, and 11 (5.5%) in the control group had a family history of early death. In our study, the reason for the high rate of a family history of early death may be the inclusion of questions about siblings of the parents and their children. Therefore, we think that an extended family history of early sibling death may be an important warning sign for the diagnosis of immunodeficient patients.

Worldwide, the mean duration of diagnostic delay between the onset of symptoms and diagnosis in PID diseases is 4.08 years. The biggest factor in an 8–10-year delay in the diagnosis of PID diseases after the onset of symptoms was the low level of physician awareness of these diseases (9). In our study, the mean duration of diagnostic delay for the PID and SID groups was 21 months, with no significant difference between the groups. Given that a delay in diagnosis can significantly increase morbidity and mortality, we may conclude that the index of suspicion for PID on the part of physicians in our region is similar to that in other centers.

In the literature, PID diseases have been reported to be more common in males than in females (6). The predominance of males results not from PIDs inherited in an autosomal recessive manner, but from X-linked PIDs. In our study of PID patients, 49% were female and 51% were male, with no statistically significant difference. We consider that this result was due to the high rate of consanguineous marriages in our study. In addition, the predominance of male gender in SID group could be due to the characteristics of the patients involved in our study.

Antibody deficiencies are the most common subtype of PID (16); they were also the most common PID group in our study. This is consistent with the European Society for Immunodeficiencies and the JMF databases, and our results are consistent with previous study results. However, given that the incidence of allergic, autoimmune, and hematological diseases, as well as the incidence of malignancies are high among PID patients, the medical history should be scrutinized in this respect (17). Studies suggest the need for some additional warning signs to facilitate early diagnosis in such patients (18, 19). In our study, the rates of the rheumatic diseases and malignancy in the PID and SID groups were significantly higher than in the control group. Patients with immunodeficiencies may present with infectious diseases, and also with immune dysregulation diseases and malignancies; we consider that these diseases should also be considered as warning signs of immunodeficiency.

The limitation of this study is that some of our patients are a few months old and the JMF warning signs are not to be highly specific in this population naturally. We comment that it should be considered the warning signs are specific after infancy.

In conclusion, early diagnosis of PID will allow effective treatment of these diseases. We agree that the 10 warning signs of PID diseases defined by the JMF are important for the early diagnosis of PID. From our study results, a family history of parental consanguinity or tuberculosis may also be warning signs of PID, and a history of chronic diarrhea should be included. Studies from different immunology centers may clarify these additions. This approach will allow early diagnosis of PID and, thus, early and effective treatment, which will allow patients to reach adulthood before the development of organ injury.

## Data Availability Statement

The original contributions presented in the study are included in the article/supplementary material. Further inquiries can be directed to the corresponding author.

## Ethics Statement

The studies involving human participants were reviewed and approved by Necmettin Erbakan University Meram Medical School Ethics Committee. Written informed consent to participate in this study was provided by the participants’ legal guardian/next of kin.

## Author Contributions

F-CE, YG, IR, SK, SG, and AY implemented the study and collected the data. F-CE, IR, S-NG, and AY wrote the manuscript. SK and S-NG analyzed the data. All authors participated in the design and interpretation of the studies, analysis of the data and review of the manuscript. All authors contributed to the article and approved the submitted version.

## Conflict of Interest

The authors declare that the research was conducted in the absence of any commercial or financial relationships that could be construed as a potential conflict of interest.

## Publisher’s Note

All claims expressed in this article are solely those of the authors and do not necessarily represent those of their affiliated organizations, or those of the publisher, the editors and the reviewers. Any product that may be evaluated in this article, or claim that may be made by its manufacturer, is not guaranteed or endorsed by the publisher.
